# Early *in vitro* evidence indicates that deacetylated sialic acids modulate multi-drug resistance in colon and lung cancers *via* breast cancer resistance protein

**DOI:** 10.3389/fonc.2023.1145333

**Published:** 2023-06-12

**Authors:** Isaac Tuffour, Setor Amuzu, Hala Bayoumi, Iram Surtaj, Colin Parrish, Rachel Willand-Charnley

**Affiliations:** ^1^Department of Chemistry and Biochemistry, South Dakota State University, Brookings, SD, United States; ^2^Department of Human Genetics, McGill University, Montreal, QC, Canada; ^3^Department of Medical Sciences, American University of Iraq, Sulaimani, Iraq; ^4^Baker Institute for Animal Health, Department of Microbiology and Immunology College of Veterinary Medicine, Cornell University, Ithaca, NY, United States

**Keywords:** breast cancer resistance protein, sialic acid, O-acetyl sialic acid, cancer, multidrug resistance

## Abstract

Cancers utilize sugar residues to engage in multidrug resistance. The underlying mechanism of action involving glycans, specifically the glycan sialic acid (Sia) and its various functional group alterations, has not been explored. ATP-binding cassette (ABC) transporter proteins, key proteins utilized by cancers to engage in multidrug resistant (MDR) pathways, contain Sias in their extracellular domains. The core structure of Sia can contain a variety of functional groups, including O-acetylation on the C6 tail. Modulating the expression of acetylated-Sias on Breast Cancer Resistance Protein (BCRP), a significant ABC transporter implicated in MDR, in lung and colon cancer cells directly impacted the ability of cancer cells to either retain or efflux chemotherapeutics. *Via* CRISPR-Cas-9 gene editing, acetylation was modulated by the removal of CAS1 Domain-containing protein (CASD1) and Sialate O-Acetyl esterase (SIAE) genes. Using western blot, immunofluorescence, gene expression, and drug sensitivity analysis, we confirmed that deacetylated Sias regulated a MDR pathway in colon and lung cancer in early *in vitro* models. When deacetylated Sias were expressed on BCRP, colon and lung cancer cells were able to export high levels of BCRP to the cell’s surface, resulting in an increased BCRP efflux activity, reduced sensitivity to the anticancer drug Mitoxantrone, and high proliferation relative to control cells. These observations correlated with increased levels of cell survival proteins, BcL-2 and PARP1. Further studies also implicated the lysosomal pathway for the observed variation in BCRP levels among the cell variants. *RNASeq* data analysis of clinical samples revealed higher *CASD1* expression as a favorable marker of survival in lung adenocarcinoma. Collectively, our findings indicate that deacetylated Sia is utilized by colon and lung cancers to engage in MDR *via* overexpression and efflux action of BCRP.

## Introduction

A variety of current chemotherapeutic treatments are compromised by multidrug resistance (MDR), a refractory mechanism, resulting in therapeutic failure and extended treatments with second or third-line therapies. Strikingly, MDR is responsible for 90% of deaths in cancer patients receiving conventional or novel-targeted therapeutics (1). It is therefore imperative to intensify research efforts to prevent and understand MDR pathways. Cancers employ MDR pathways *via* a multitude of mechanisms including mutations in drug targets, altered drug metabolism, diminished apoptosis (cell death) signaling, and reduced drug accumulation (2), with reduced drug accumulation accounting for the majority of chemotherapy resistance (3–5). This particular MDR mechanism occurs through the overexpression of a group of heavily glycosylated transmembrane proteins referred to as ATP-binding cassette (ABC) transporters. ABC transporters, known as efflux pumps, belong to a large protein superfamily comprising of 48 members that facilitate the energy-dependent extrusion of a broad range of compounds against a concentration gradient (6). The major ABC transporters implicated in clinical MDR include permeability-glycoprotein (P-gp), the multidrug resistance protein 1 (MRP1), and the breast cancer resistance protein (BCRP) (7, 8).

In this study, attention was given to BCRP due to its broad substrate specificity. Human BCRP is the second member of the ATP binding cassette sub-family G (ABCG2), encoded by the *ABCG2* gene that is located on chromosome 4q22 (9). Physiologically, it is highly expressed in placenta, brain micro-vessel endothelium, mammary gland, colon, liver, small intestine, biliary tract, ovary, testis, kidney, and hematopoietic stem cells; contributing to the pharmacokinetics of drugs and endogenous compounds as well as protecting tissues from toxic xenobiotics (10–12). It actively transports a broad spectrum of conventional anticancer agents, including doxorubicin, mitoxantrone, topotecan, methotrexate, and daunorubicin as well as novel targeted molecules such as the tyrosine kinase inhibitors, imatinib and gefitinib (13, 14). BCRP is overexpressed in many malignancies and contributes to poor chemotherapeutic response in acute myelogenous leukemia (AML), chronic myeloid leukemia (CML), pancreatic ductal adenocarcinomas, non-small lung cancer cells (NSCLC), and other solid tumors (10). Existing therapeutic strategies aimed at overcoming BCRP mediated MDR include various pharmacological classes of inhibitors such as tyrosine kinase inhibitors (TKIs), HCV protease inhibitors, antifungal azoles, and immune suppressants (15–18). Although these inhibitors have shown MDR reversal activity in pre-clinical studies, they have yielded limited clinical success due to toxicity and off-target drug interactions when co-administered with additional anticancer drugs, prompting the need for alternative clinically relevant therapeutics (19–21). Several mechanisms including transcriptional (promoter activation by cis-acting elements and miRNAs targeting BCRP/ABCG2), epigenetic (CpG island hypomethylation and histone hypermethylation), post-translational (e.g., BCRP phosphorylation by Pim-1 kinase) and glycans have been reported to modulate BCRP expression (22–25). The role of sialic acid and its associated functional group alterations in MDR remained unexplored.

Glycans are present on asparagine residues (N-linked recognition sites) in specific extracellular loops connecting the transmembrane helices of the transporter proteins (9, 26, 27). Although prior studies have reported a positive correlation between glycosylation status of BCRP and drug resistance (poor therapeutic outcomes) in cancers, for instance (28) reported significant reversal of MDR phenotypes in ovarian and colorectal cancers following chemical inhibition of BCRP glycosylation, Sia remained uninvestigated. Furthermore, the functional checkpoints of BCRP revealed that mutagenic substitution of asparagine 596, the crucial glycosylation site on BCRP, with glutamine disrupted N-linked glycosylation, interfered with localization, function of BCRP, and sensitized hitherto MDR cancer cells to chemotherapeutics (29). Lastly, lectin binding experiments revealed branched oligosaccharide chains capped with either α-2,6 or α-2,3 linkage of sialic acids (30–32).

**Figure 1:**
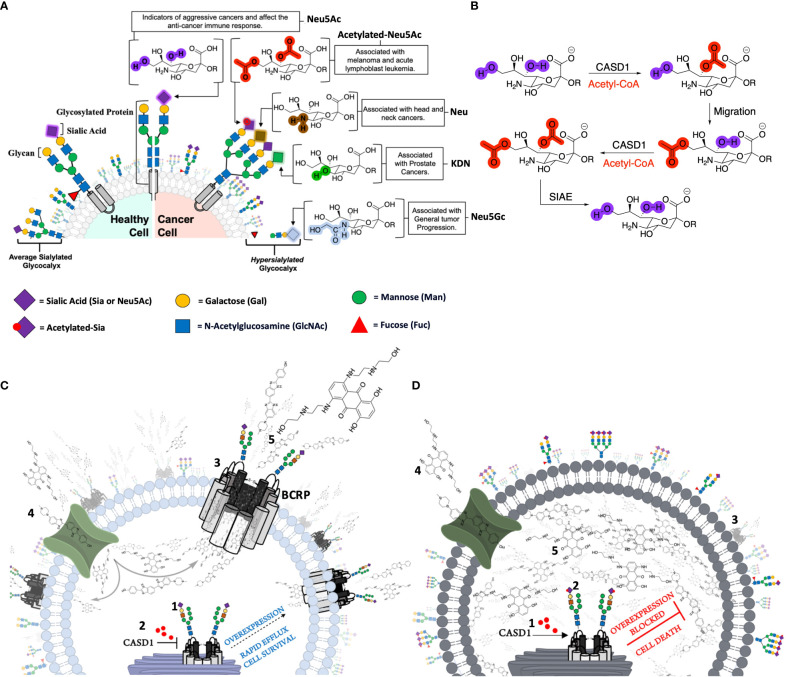
The contribution of canonical and non-canonical forms of Sialic acids in cancer and cancer related pathways. **(A)** Healthy cells are covered with an averagely sialylated glycocalyx and present moderate levels of Sialic acids (Sia or Neu 5Ac) on glycosylated proteins. Malignant or cancer cells on the other hand have an altered Sialome, expressing high levels of both canonical (unmodified) and non-canonical forms of Sia as a mechanism for survival. Overexpression of Neu 5Ac enables cancer cells to evade immune-mediated death and serves as an indicator of aggressiveness. Neu5Gc overexpression is also associated with general tumor progression. Non-canonical Sias including the Deacetylated (Neu) and Deaminated (KDN) forms are associated with Head/Neck and Prostate cancers respectively. Melanomas and Acute Lymphoblast Leukemias (ALL) distinctly express high levels of Acetylated Neu5Ac. **(B)** CASD1 and SIAE enzymes modulate 9-O and 7,9-O acetylation of Sia (Neu 5Ac). CASD1 adds acetyl functional groups, via an Acetyl CoA donor, to the seventh carbon of Sia, from which it migrates to the ninth carbon (Neu 5,9Ac2) under physiological conditions. This facilitates the addition of new acetyl group to the seventh carbon by CASD1 yielding Neu 5,7,9Ac3. SIAE can remove acetyl functional groups, regenerating the canonical form of Sia (Neu 5Ac) **(C)** Sia (purple diamonds) is added to the glycan chain on BCRP in the golgi by Sialyltransferases (2) In the absence of CASD1 (i.e., CASD1 Knockout), no acetyl groups (red circles) are added to the sialylated transporter (3) Majority of deacetylated transporters are successfully trafficked to the cell surface (4) Chemotherapeutics and BCRP substrates targeted at cells, permeate into the cytoplasm (5) The high expression levels of BCRP at the cell surface confers a multidrug resistance phenotype as large amounts of chemotherapeutics are rapidly pumped out enabling the cell to survive. **(D)** CASD1 adds acetyl groups (red circles) to (2) sialylated glycan chain on transporter (3) The majority of the transporter with acetyl group modification is transported to the lysosome for degradation (4) Chemotherapeutics and BCRP substrates targeted at cells, permeate into the cytoplasm (5) Due to the low expression levels of BCRP at the cell surface, only a small fraction of chemotherapeutics is pumped out of the cell with high amount accumulated in the cytoplasm exerting prolonged pharmacological effect leading to cell death (apoptosis). *For simplicity, the authors have deliberately excluded sialylated O-glycans and gangliosides.

Sialic acids (Sia e.g., N-acetylneuraminic acid [Neu5Ac]) are a family of 9-carbon sugars found as terminal residues on cell surface glycoproteins, glycolipids, and gangliosides (33–35). Aberrant expression of sialic acids on death receptors TNFR1 and FasR has been widely reported to inhibit cell death and promote drug resistant phenotypes in ovarian, pancreatic and colon cancer cells (36, 37). Ma et al. (38) also reported increased levels of ST3GAL5 and ST8SIA4 sialyltransferases in drug resistant human acute myeloid leukemia (AML). They further identified that these alterations regulated the activity of PI3K/Akt signaling and correlated with the overexpression of ABC transport proteins, suggesting a strong correlation between glycan sialylation and MDR thus warranted further exploration. The hydroxyl groups associated with Sia may be further chemically modified with various functional groups including acetyl, lactyl, methyl, sulfate, or phosphate groups (34). Acetylated Sias are the most clinically studied modification. Of specific interest are the modifications to the C6 hydroxylated tail. Modulation of the acetyl functional group C6 tail are mediated by two enzymes, CAS 1 Domain-containing protein (CASD1) and Sialic acid acetyl esterase (SIAE) respectively (35). Driven by our recent discovery that the overexpression of deacetylated-Sia on colon and lung cancers results in immune evasion, of Natural Killer (NK) mediated cytotoxicity *via* the Siglec-Sia pathway (35), we began turning our attention to additional mechanistic pathways undermining the effective prevention of cancers involving glycosylation, specifically deacetyl-Sias. Although Sias have been implicated in several cancer hallmarks including metastasis and invasion (39) (**Figure 1A**), the role acetyl functional group modulation on Sia play in MDR remained unexplored. As a result, and based on our prior work, we hypothesized that increasing the expression of deacetylated Sias on BCRP would modulate MDR *via* BCRP expression on the cell’s surface. As such, we investigated BCRP expression and function as well as survival in control, CASD1 knockout cells that lack acetylated Sia, and SIAE knockout cells that over-express acetylated Sia in lung and colon cancer cell line.

## Materials and methods

### Scientific rigor

All reagents/antibodies/cell lines have been selected based on published figures and purchased from companies that provide validation. Experiments were performed in technical triplicates and then biological triplicates. We used t-tests or ANOVA followed by Tukey post-tests for multiple comparisons using GraphPad Prism 8 (San Diego, CA). Data, as seen below, is always presented as mean ± standard deviation with P < 0.05 indicating significance.

### Reagents

Fetal bovine serum (FBS) and penicillin/streptomycin (P/S) were obtained from Corning Incorporated (Corning, NY). The GAPDH loading control monoclonal antibody (ref AM4300, lot #: 00939504), Alexa Fluor 488 conjugated goat anti-mouse IgG cross-adsorbed secondary antibody (2 mg/mL, Cat #A32723), PowerUp SYBR green PCR master mix (Cat # A25741) and paraformaldehyde were obtained from Thermo Fisher Scientific Inc (Rockford, IL). Anti-ABCG2 antibodies, BCRP/ABCG2 (Cat #EPR20080, lot #: 3026758) and clone BXP-21 (Cat #MAB4146) were obtained from Abcam (Waltham, MA) and Millipore (Billerica, MA) respectively. IRDye Goat anti-mouse and anti-rabbit IgG secondary antibody (lot#: C90130-02) were obtained from LI-COR (Lincoln, NE). Mitoxantrone, dimethyl sulfoxide (DMSO), 3-(4,5-dimethylthiazolyl)-2,5-diphenyltetrazolium bromide (MTT), 4′,6-diamidino-2-phenylindole (DAPI), Ko143, Bafilomycin A1, and Triton X-100 were purchased from Sigma Chemical Co (St. Louis, MO). All other chemicals used were of analytical grade.

### Cell lines and cell culture

A549 cells were cultured in Dulbecco modified Eagle medium (Corning) supplemented with 10% FBS and 1% P/S (Corning). HCT 116 cells were cultured in RPMI 1640 medium (Corning) supplemented with 10% FBS and 1% P/S. All cell lines were originally purchased from American Type Culture Collection (Rockville, MD). Cell Dissociation Buffer (Gibco, Waltham, MA) was exclusively used for passaging cells.

CASD1 and SIAE knockout A549 cell lines were obtained from Dr. Colin Parrish (Cornell University, Ithaca, NY). CRISPR-Cas9 stably expressing cells and the third-generation lentiviral system were gifted from Dr. Michael Bassik (Stanford University, Stanford, CA). HEK 293/PcDNA 3.1 cells were a generous gift from Dr. Surtaj Iram (American University of Iraq, Sulaimani, Iraq). CRISPR-Cas9 editing of CASD1 and SIAE in HCT 116 and A549 cells was previously published (35, 40). Briefly, paired Cas9 plasmids targeting adjacent sites in early exons of *CASD1* and *SIAE* were transfected using TransIT-X2 (Mirus Bio LLC, Madison, WI). Transfected cells were selected with puromycin, and single cell clones screened with a specific virolectin recognizing 9-O-acetyl Sia (PToV-P4 HE-Fc) (35, 40, 41). Full sequencing was used to confirm loss of *CASD1* and *SIAE* function in both alleles. qPCR and western blot analysis were also performed to confirm knockout efficiencies.

### Generation of BCRP overexpressing HEK 293 cell line

A BCRP (ABCG2)-overexpressing cell line was developed and used as a positive control for efflux studies. Briefly, HEK 293 Wildtype cells were plated in 6 well plate (Corning, NY) at a density of 5x10^5^ cells/ml and cultured until 80% confluency was attained. Cells were then transiently transfected with 2 µg PcDNA 3.1 plasmid containing Human ABCG2 cDNA ORF clone (GenScript Biotech, NJ). Transfection was carried out with Lipofectamine 3000 (Invitrogen, MA) in Opti-MEM medium (Gibco, MD), according to manufacturer’s protocol (42). ABCG2-expressing stable cells were selected by treating cells with 0.8mg/ml G418 for up to a week. A G418 (Geneticin) (Gibco, MA) kill curve was performed prior to transfection on HEK 293 Wildtype cells to select appropriate G418 concentration for stable cell line generation. Western blot analysis was used to confirm stable transfection.

### Western blot analysis

Cell lysates were prepared in RIPA buffer (Thermo-Fisher Scientific, MA) supplemented with Protease and Phosphatase Inhibitor (ThermoFisher Scientific, MA). Protein concentration was determined using Pierce BCA Protein Assay (ThermoFisher Scientific, MA). Cell lysates (20-30 µg protein) were electrophoresed on 4-12% Criterion TGX Precast gels (BioRad, CA) and transferred onto Trans-Blot turbo nitrocellulose membranes (BioRad, CA). Membranes were blocked with Intercept blocking buffer (LI-COR, NE) for 1 h at room temperature and incubated overnight at 4°C with monoclonal anti-BCRP antibody (Abcam, MA), anti-GAPDH antibody (Thermo Fisher Scientific, IL), anti-Bcl2 antibody (Thermo Fisher Scientific, IL) and anti-PARP1 antibody (Thermo Fisher Scientific, IL) at 1:500, 1:2000, 1:1000 and 1:1000 dilutions in blocking buffer respectively. Secondary antibody incubation (1:1000 dilution in PBS containing 0.1%Tween 20) was performed using goat anti-mouse IRDye secondary antibody (LiCOR, NE) and goat anti-rabbit IRDye secondary (Li-COR, NE) for 1 h at room temperature. Target proteins were detected using the Odyssey CLx Imager (LiCOR, NE). For protein expression comparison, protein band density was analyzed using Image StudioLite (LI-COR, NE) software and corrected for uneven sample loading and transfer using GAPDH as the loading control. For expression levels of Cleaved PARP1 determination, cells were challenged with 0.5µg/ml Mitoxantrone for 48h prior to lysate preparation. This same approach was used to investigate the effect of the lysosomal enzyme inhibitor, Bafilomycin A1 (BMA), on BCRP protein expression levels. Cells (4 x 10^5^ cell/ml) were challenged with 100nM BMA for 2h prior to lysate preparation using a previously established protocol (29).

### Immunofluorescence assay

Cellular expression of BCRP was determined by immunofluorescence microscopy as previously described (43). In brief, HCT 116, A549 and HEK/BCRP cells (2 × 10^4^ cells per well) were grown on glass coverslip. Cells were then fixed in 4% paraformaldehyde and permeabilized with 0.5% Triton X-100. Slides were blocked in a buffer containing 0.01% goat serum, 0.01% saponin, and 0.05% glycine in PBS for 1 h. After incubation with 1:20 dilution of human ABCG2 antibody BXP-21 (Millipore, MA) in PBS (containing 0.1% BSA) overnight at 4°C, cells were incubated with 1:200 dilution Alexa Fluor 488 conjugated goat anti-mouse IgG cross-adsorbed secondary antibody (Thermo Fischer, IL) in PBS (containing 0.1% BSA) in the dark for 1 h. DAPI (Sigma, MO) (1µg/mL final concentration) was used to stain nuclei of cells. BioTek Cytation Live Cell imager (BioTek, WA) was used to collect immunofluorescence images.

### Hoechst 33342 accumulation assay

Intracellular fluorescence of Hoechst 33342 was analyzed with a fluorescence microscope (Carl Zeiss, Goettingen, Germany) as described previously (44). Briefly, cells were cultured in a 6-well plate (Corning, NY) containing poly-L lysine coated glass cover slips overnight at a density of 5x10^5^ cells/ml. Cells were exposed to 1μM Hoechst 33342 in the presence or absence of 1μM Ko143. Cells were then washed twice with ice-cold PBS, covered with HEPES buffer and fluorescence images were measured. Image StudioLite (LI-COR, NE) software was used to quantify fluorescence intensity (Hoechst 33342 accumulation).

### Cell viability assay

Differential sensitivity of cells to Mitoxantrone was measured using the MTT (3-(4,5-dimethylthiazolyl)-2,5-diphenyltetrazolium bromide) colorimetric assay as described previously (45). Briefly, cells were plated at a density of 2x10^5^ cells/ml in a 96 well plate (Corning, NY) and challenged with varying concentrations (0-5 µg/ml) of Mitoxantrone for 48 h in the presence or absence of the BCRP inhibitor, Ko143 (1µM). MTT solution (5 mg/ml) was then added to each well and cells further incubated for 4 h at 37°C. The amount of formazan produced was measured at a wavelength of 570 nm with a microplate reader (BioTek Cytation 3, WA). Absorbance values were recorded and used to evaluate the percentage cell viability. GraphPad Prism 8 software was used to evaluate the individual IC_50_ values, following which fold resistance (ratio of IC_50_ value for each cell line to IC_50_ of its corresponding Wildtype cell line) was estimated.

### Cell proliferation assay

Growth rate of cells was measured and compared *via* the method described by Barnard et al. (40). Briefly, cells were plated 2x10^5^ cells/ml in a 96 well plate (Corning, NY) overnight. The cells were subsequently harvested, stained with trypan blue and counted every day for up to 4 days with an automated cell counter (BioRad, CA). Culture medium was replaced with fresh culture medium after the second day for days 3 and 4 of the experiments.

### mRNA expression analysis

Total RNA was extracted from each cell variant using Direct-zol RNA mini-prep kit (Zymo Research Corporation, Irvine, CA), according to the manufacturer’s instructions. First-strand cDNA was prepared from the extracted RNA in a reverse transcriptase reaction with a High-Capacity cDNA Archive Kit (Applied Biosystems, Foster City, CA) and random hexamers as a primer, according to the manufacturer’s protocol in an iCycler™ thermal cycler (Bio-Rad Laboratories Inc., Hercules, CA). cDNA concentration and purity were measured with a Nanodrop 2000 spectrophotometer (Thermo Scientific, USA). Using the primer sets (Integrated DNA Technologies, Coralville, IA) shown below, relative BCRP gene expression was further investigated by quantitative real-time PCR.

ABCG2: 5’-GATCTCTACCCTGGGGCTTGTGGA-3’; 5’-TGTGCAACAGTGTGATGGCAAGGGA-3’

GAPDH: 5’-ACTGCCAACGTGTCAGTGGTGGACCTGA-3’; 5’-GGCTGGTGGTCCAGGGGTCTTACTCCTT-3′.

Briefly, each cDNA sample (1 µl) was amplified with 10 µl of Thermo Scientific PowerUp SYBR Green qPCR Master Mix and 1 µM of each primer. Amplification was performed in a Quant Studio 3 Real Time PCR system (Applied Biosystems, Foster City, USA) with the following parameters: UDG activation at 50°C, Activation (Dual-Lock DNA polymerase) at 95°C for 2 min followed by 40 cycles of denaturation at 95 °C for 15 s, annealing/extension at 60°C for 1 min. The relative expression levels of ABCG2 in each sample (normalized to that of GAPDH) were determined using 2^−ΔΔCt^ method. RT-qPCR experiments were repeated three times.

### Expression of *CASD1* and *SIAE* in clinical samples

To investigate the potential prognostic value of *CASD1* and *SIAE*, we performed survival analysis of *CASD1* and *SIAE* mRNA expression and overall survival in clinical lung adenocarcinoma (LUAD) samples (n=501) and clinical colon adenocarcinoma (COAD) samples (n=374) from the TCGA PanCancer Atlas study (46). Survival data and gene expression data were downloaded *via* cBioPortal (https://www.cbioportal.org/) (47, 48). Gene expression data downloaded for our analysis were *mRNA Expression, RSEM (Batch normalized from Illumina HiSeq_RNASeqV2)*. Survival analysis and visualization were performed with R programming language using *ggsurvfit* and *survival* (49) packages. Kaplan-Meier plots were generated to visually compare survival across gene expression groups and cancer types. Samples were split into two groups, high and low, based on the median expression per gene (high: >=median, low: <median). Overall survival status was coded 1 for deceased and 0 for censored, overall survival time was expressed in months, and gene (*CASD1* or *SIAE*) expression status was coded 1 for low and 2 for high. Univariable Cox proportional hazard regression models were fit using survival outcome (based on overall survival status and overall survival time) as response variable and gene expression status as explanatory variable. The Cox proportional hazard regression hazard ratio (HR), 95% confidence intervals (CI) and log-rank p value were reported in each Kaplan-Meier plot. Statistical significance was set at log-rank p value < 0.05.

## Results

### Generation of BCRP/ABCG2 overexpressing HEK 293 cells

To develop a stable BCRP-overexpressing cell line to serve as a positive control for functional studies, we first constructed a Geneticin (G418) kill curve to determine appropriate G418 concentration for stable cell line selection. The results revealed that 0.8mg/ml G418 inhibited 99% cell growth (**Figure S1A**). HEK 293 Wildtype were then transiently transfected with a PcDNA 3.1 vector containing Human ABCG2 cDNA ORF clone and selected for stably transfected cells by applying 0.8mg/ml G418 pressure for a week. Results of transfection revealed high BCRP/ABCG2 protein expression in transfected cells (HEK 293/BCRP) compared to the Wildtype cell line (HEK 293 WT) and cell transfected with an empty vector (HEK 293/PcDNA3.1) (**Figure S1B**). Also, the generated HEK 293/BCRP cell line was functionally characterized by comparing it to HEK 293 WT in terms of their response or sensitivity to the chemotherapeutic drug, Mitoxantrone. Our results showed significantly increased cell viability (**Figure S1C**) and 5-fold increased resistance (**Supplementary Table 1**) in transfected cell compared to wildtype cells.

### Sialate O-acetyltansferase knockout upregulates BCRP/ABCG2 expression

**Figure 2:**
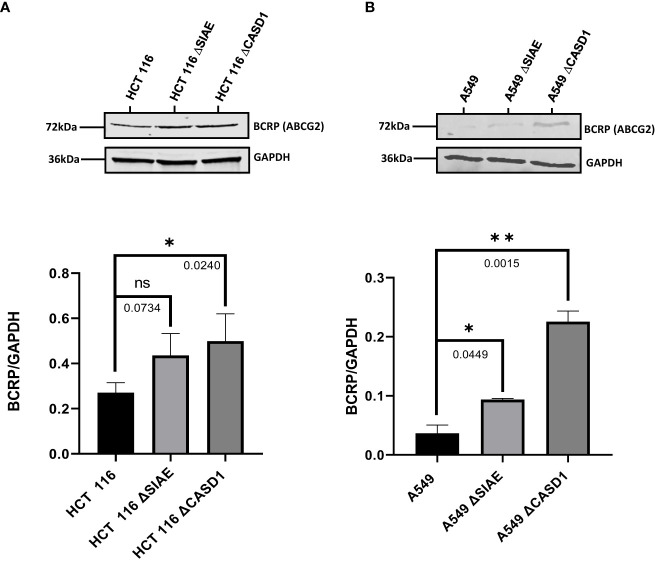
BCRP (ABCG2) expression in Wild type, Knockout (CASD1 and SIAE) cell lines. Western blot analysis was performed on whole cell lysate using BCRP-specific antibodies. GAPDH was used as loading control **(A)** Immunoblot analysis of whole cell lysates prepared from HCT 116 cell lines. Below is normalized Protein band density. **(B)** Immunoblot analysis of whole cell lysates prepared from A549 cell lines. Below is normalized Protein band density. One-way ANOVA with Tukey’s posttest for multiple comparison, *P < 0.05, **P < 0.01.

**Figure 3:**
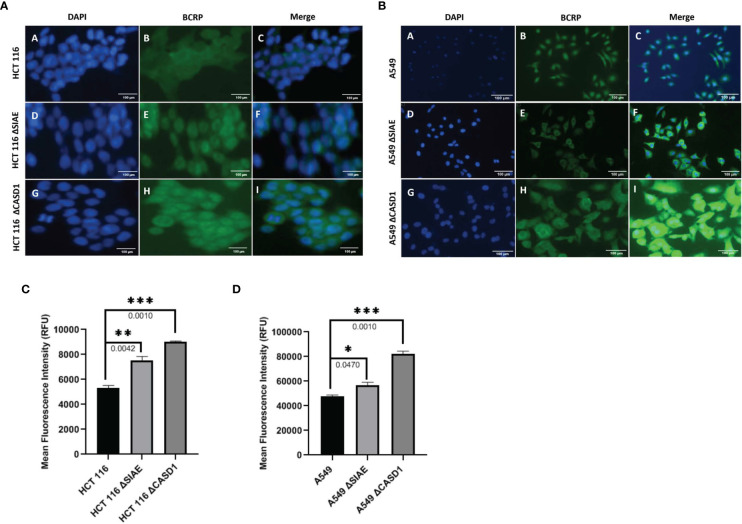
BCRP (ABCG2) expression in Wild type, Knockout (CASD1 and SIAE) cell lines. Cells were fixed, permeabilized, treated with BCRP-specific primary antibody and Alexa-Fluor 488 conjugated secondary antibody. DAPI was used for counter staining **(A)** Immunofluorescent localization of BCRP in HCT 116 cell lines. **(B)** Immunofluorescent localization of BCRP in A549 cell lines. **(C)** BCRP Fluorescence Intensity plot for HCT 116 cell lines. **(D)** BCRP Fluorescence Intensity plot for A549 cell lines. One-way ANOVA with Tukey’s posttest for multiple comparison, *P < 0.05, **P < 0.01 and ***P ≤ 0.001.

To determine the effect of O-acetyl modification of Sia on BCRP, western blot analysis and immunofluorescence assay were performed to compare BCRP protein expression in Wildtype HCT 116 and A549 cell lines to those without O-acetyl Sia (CASD1 knockout) and those that express high levels of O-acetyl Sia (SIAE knockout). The results indicate that knockout cells showed high expression of BCRP (ABCG2) in both HCT 116 (**Figures 2A**, **3A, C**) and A549 (**Figures 2B**, **3B, D**) cell lines as compared to wild type cells. CASD1 knockout cells recorded the most significant overexpression of BCRP (p-values =0.0240 and 0.015 for HCT 116 and A549 cell lines respectively) followed by SIAE knockout cells (p-value = 0.0449 for A549 cell line). Also, CASD1 knockout A549 cells comparatively appeared cytologically transformed (i.e., increase in size) (**Figure 3B**).

### CASD1 knockout decreases intracellular accumulation of Hoechst 33342

**Figure 4:**
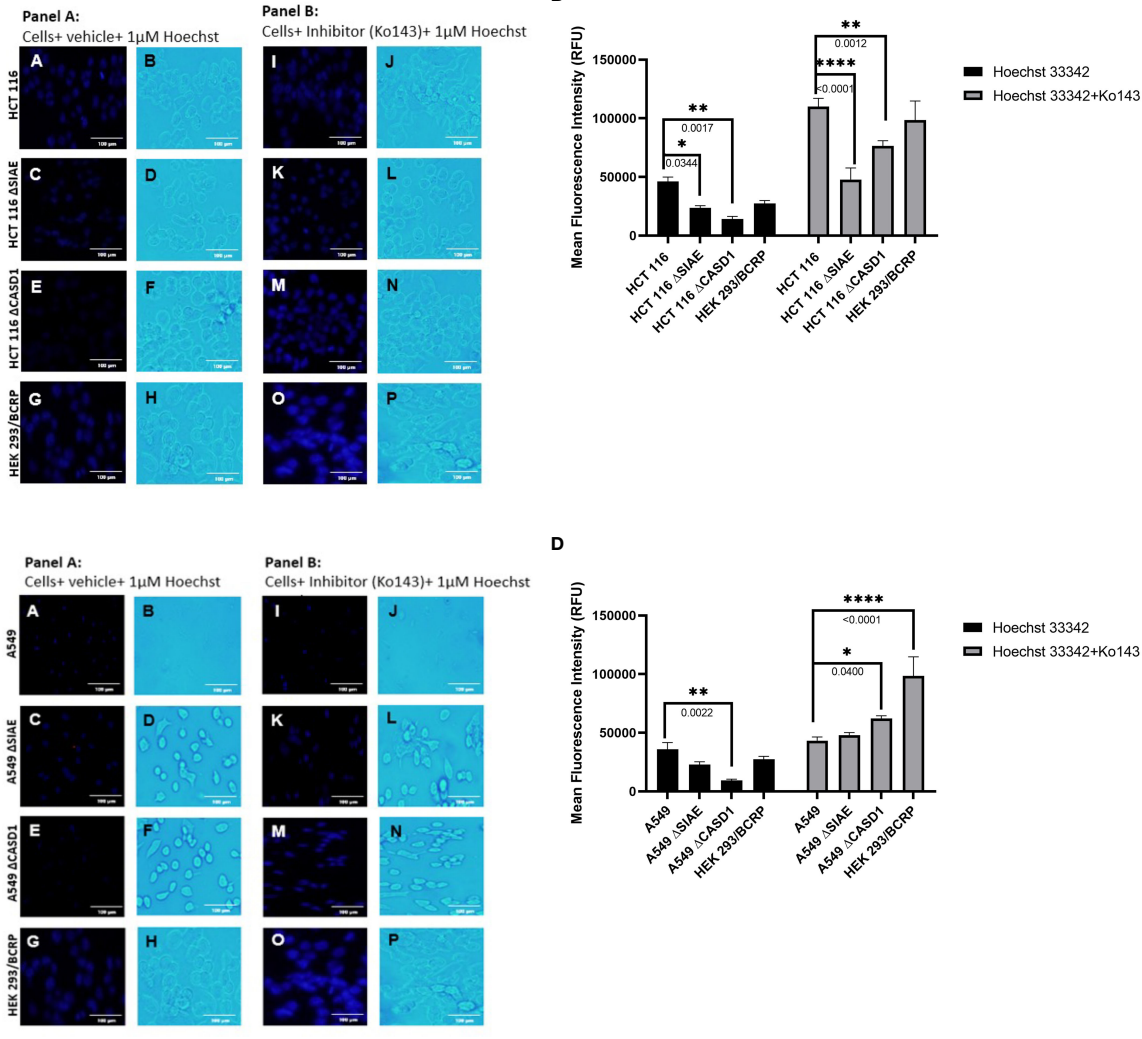
Intracellular accumulation of Hoechst 33342 in HEK and A549 Cells. Cells were treated with Hoechst 33342 in the presence or absence of Ko143 and stained with Hoechst 33342. **(A)** Fluorescent and Bright field images of HCT 116 cells. **(B)** Fold change in Hoechst 33342 accumulation in HCT 116 cells. **(C)** Fluorescent and Bright field images of A549 cells. **(D)** Fold change in Hoechst 33342 accumulation in A549 cells. One-way ANOVA with post hoc Tukey’s test. *P < 0.05, **P < 0.01, and ****P <0.0001.

To investigate the effect of O-acetylated Sia modifications on BCRP (ABCG2) function, BCRP fluorescent substrate, Hoechst 33342 was used to assess transport (efflux) competence of wild type and knockout cell lines. To further prove BCRP’s involvement in this efflux phenomenon, potent BCRP inhibitor, Ko143 was used to block efflux function. Our data shows generally high fluorescent intensities for Ko143-inhibited cell lines compared to control (vehicle-treated) cell lines (**Figures 4B, D**). Also, we observed reduced accumulation of Hoechst 33342 (i.e., high efflux) in knockout cell lines compared to wild type cell lines (**Figure 4B**), and (**Figure 4D**). Comparatively, CASD1 knockout cells recorded the most significant reduction in Hoechst fluorescence intensity in HCT 116 cell lines (i.e., Panel A of **Figures 4A**, **B** (p-value = 0.0344) and A549 cell lines (i.e., Panel A of **Figures 4C**, **D** (p-value = 0.0022)

### CASD1 knockout confers resistance to mitoxantrone

**Figure 5:**
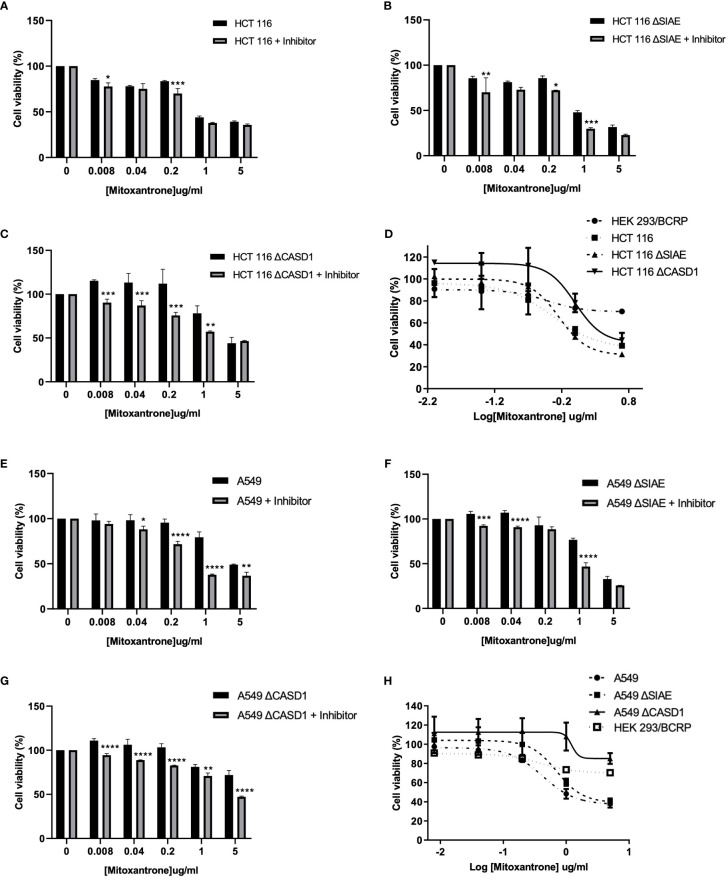
Cytotoxic effect of Mitoxantrone on HCT 116 and A549 cells. Cells were treated with Mitoxantrone (0-5ug/ml) in the presence or absence of 1uM Ko143. Dose-response of the effect of Mitoxantrone on **(A)** HCT 116 Wildtype cells **(B)** HCT 116 SIAE knockout cells **(C)** HCT 116 CASD1 knockout cells **(D)** Comparative cell viability profiles. Data reported as mean + sd of three independent experiments. Two-way ANOVA with Tukey’s posttest for multiple comparison, *P ˂ 0.05. Cytotoxic effect of Mitoxantrone on A549 cells. Cells were treated with Mitoxantrone (0-5ug/ml) in the presence or absence of 1uM Ko143. Doseresponse of the effect of Mitoxantrone on **(E)** A549 Wildtype cells **(F)** A549 SIAE knockout cells **(G)** A549 CASD1 knockout cells **(H)** Comparative cell viability profiles. Data reported as mean + sd of three independent experiments. Two-way ANOVA with Tukey’s posttest for multiple comparison, *P < 0.05, **P < 0.01, ***P < 0.001 and ****P < 0.0001.

To investigate the effect of O-acetyl Sia on cancer cell response to the chemotherapeutic drug, Mitoxantrone, the MTT (tetrazolium-based) cell viability assay was performed. Following exposure to varying concentrations (0-5µg/ml) of Mitoxantrone for 48h in the presence or absence of BCRP inhibitor (Ko143), the result revealed reduced cell viability in all cancer lines in the presence of Ko143 inhibitor as compared to vehicle treated (uninhibited) cells (**Figures 5A–C**, **5E–G**). The results further showed a reduced sensitivity/response in CASD1 knockout cell lines of both cancer cells as compared to the Wild types and SIAE knockout cells. This is inferred from the IC_50_ values of 0.512, 0.658 and 1.009 µg/ml recorded for HCT 116 Wildtype, HCT 116 SIAE knockout and HCT 116 CASD1 knockout cell lines respectively (**Table 1**; **Figure 5D**). A549 Wildtype, A549 SIAE knockout and A549 CASD1 knockout cell lines also recorded IC_50_ values of 0.423, 0.710 and 1.226 µg/ml respectively (**Table 1**; **Figure 5H**). In effect, CASD1 knockouts of both cancer cells exhibited significantly higher resistance towards Mitoxantrone (i.e., ~2-fold resistance and 3-fold resistance for HCT 116 and A549 cell lines respectively when compared to their Wildtype cells) (**Table 1**)

**Table 1:** Effect of Acetyl Sia on Cellular response to Mitoxantrone.

Cell line	IC_50_ (µg/ml)	
	Mitoxantrone	Fold Resistance
HCT 116	0.512 + 0.070	1.00
HCT 116 SIAE Knockout	0.658 + 0.012	1.28
HCT 116 CASD1 Knockout	1.009 + 0.017*	1.97
HEK 293/BCRP	1.013 + 0.012*	1.96
		
A549	0.423 + 0.132	1.00
A549 SIAE Knockout	0.710 + 0.177	1.68
A549 CASD1 Knockout	1.226 + 0.479**	2.90
HEK 293/BCRP	1.013 + 0.012**	2.39

Mean + SD of three independent experiments performed in triplicate. Fold resistance determined by dividing the IC_50_ value for each cell line by the IC50 value of Wild type.

*P < 0.05 and

**P < 0.01 significantly different from the Wild type cell line

### CASD1 knockout enhances cell proliferation

**Figure 6:**
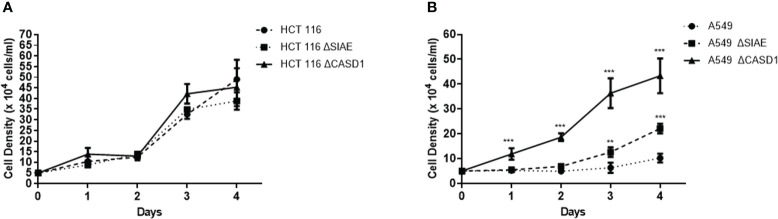
Comparative cell proliferation/growth assessment. 5X104 cells/ml was seeded and cell density was measured every 24h for 4 days **(A)** Cell growth profile for HCT 116 cell lines **(B)** Cell growth profile for A549 cell lines. Data reported as mean + sd of three independent experiments performed in triplicates. *P < 0.05, **P < 0.01 and ***P < 0.001.

To examine the association of O-acetyl Sia as well as BCRP expression in phenotypic drug resistant (aggressive) characteristics, we monitored the growth rate/cell proliferation of the wild type and knockout cells over a period of 4 days. Our results revealed significantly increased cell proliferation in the knockout cells compared to the wild type for the A549 cells (**Figure 6B**). The CASD1 knockout cell line demonstrated the highest growth rate over the 4-day period of examination followed by the SIAE knockout cell line (**Figure 6B**). A similar cell proliferation pattern was observed for HCT 116 cell lines (**Figure 6A**), however, there was no significant difference between growth rate of wild type and knockout cells.

### CASD1 knockout increases expression of cell survival proteins

**Figure 7:**
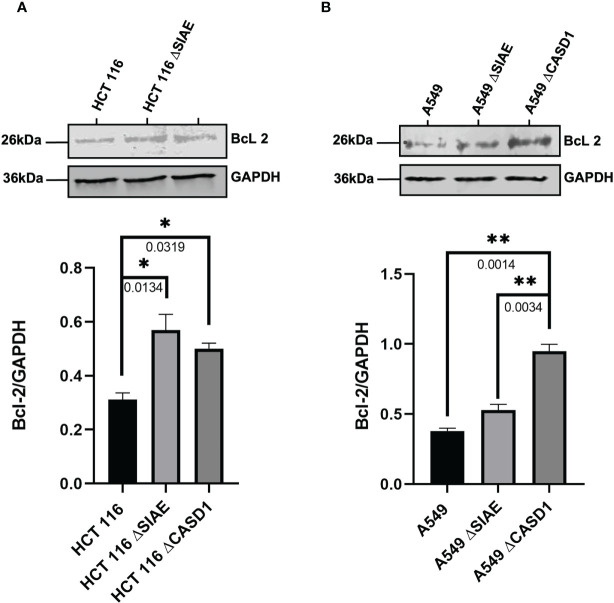
Bcl-2 Protein Expression in Wild type, Knockout (CASD1 and SIAE) cell lines. Western blot analysis was performed on whole cell lysate using BcL2-specific antibodies. GAPDH was used as loading control **(A)** Immunoblot analysis of whole cell lysates prepared from HCT 116 cell lines. Below is the normalized Protein band density **(B)** Immunoblot analysis of whole cell lysates prepared from A549 cell lines. Below is Protein band density. One-way ANOVA with Tukey’s posttest for multiple comparison, *P < 0.05 and **P < 0.01.

**Figure 8:**
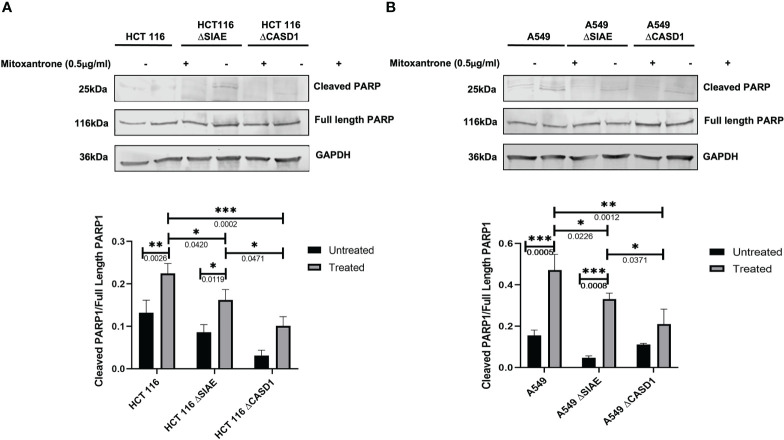
Cleaved PARP levels in Wild type, Knockout (CASD1 and SIAE) cell lines. Western blot analysis was performed on whole cell lysate using Cleaved PARP-specific antibodies. GAPDH was used as loading control **(A)** Immunoblot analysis of whole cell lysates prepared from HCT 116 cell lines. Below is the normalized Protein band density. **(B)** Immunoblot analysis of whole cell lysates prepared from A549 cell lines. Below is the normalized Protein band density. One-way ANOVA with Tukey’s posttest for multiple comparison, *P < 0.05, **P < 0.01 and ***P < 0.001.

To better understand the mechanism of CASD1 knockout-mediated cell proliferation as well the role O-acetyl Sia play in cell survival, we investigated the levels of cell survival proteins Bcl-2 and PARP 1 in the wild type and knockout cell lines. Our results showed significantly higher levels (~2 fold) of Bcl-2 expression in SIAE and CASD1 knockout HCT 116 cell lines relative to their counterpart wild type cell line (**Figure 7A**). In comparison to the wildtype cell line, the CASD1 and SIAE knockouts of A549 cell lines also recorded higher Bcl-2 protein expression; however, between the two, CASD1 knockout cell showed a much higher and significant (~2 fold) Bcl-2 protein expression (**Figure 7B**). To elucidate the protein expression levels of the 25 kDa PARP1 fragment (cleaved PARP 1) cells were incubated in the presence or absence of the apoptosis inducer, Mitoxantrone. Generally low levels of cleaved PARP1 were observed in the absence of Mitoxantrone, however, levels increased significantly in treated Wild type and SIAE knockout cell lines (**Figures 8A, B**). On the other hand, increase in Cleaved PARP 1 levels in Mitoxantrone-treated CASD1 knockouts for both HCT 116 and A549 cell lines were not significant compared its untreated counterpart. Also, CASD1 knockout cell lines expressed the lowest levels (~2 fold less) cleaved PARP1 compared wild type and SIAE knockout cell lines (**Figures 8A, B**).

### CASD1 Knockout upregulates BCRP/ABCG2 mRNA expression levels

**Figure 9:**
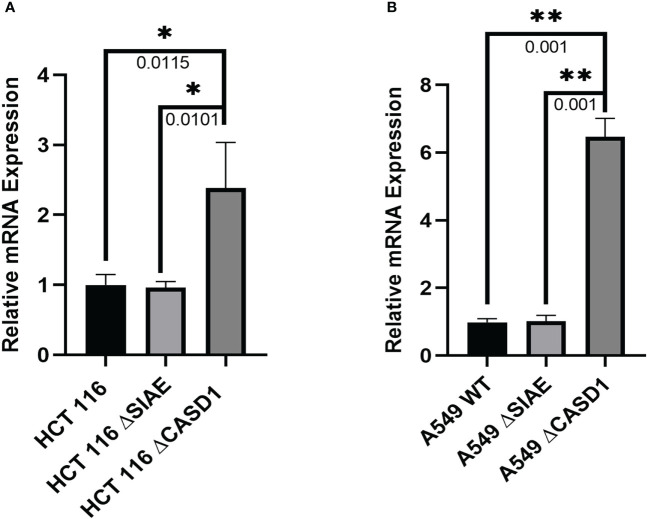
BCRP (ABCG2) mRNA expression in Wild type, Knockout (SIAE and CASD1) cell lines via qPCR. **(A)** BCRP mRNA expression profile from HCT 116 cell lines. **(B)** BCRP mRNA expression profile of A549 cell lines. One-way ANOVA with Tukey’s posttest for multiple comparison. *P < 0.05, **P < 0.01.

We explored gene expression events as a possible factor linking deacetylated Sia and BCRP protein expression by analyzing the relative expression levels of BCRP mRNA. Our results showed statistically comparable BCRP mRNA levels in Wild type and SIAE Knockout variants of both HCT 116 and A549 cells. However, significantly high expression levels were observed in CASD1 knock out cells (i.e., ˜2.5-fold and ˜ 6-fold increase in HCT 116 and A549 cells lines respectively) (**Figures 9A, B**),

### Lysosomal pathway is involved in decreased BCRP protein stability and levels

**Figure 10:**
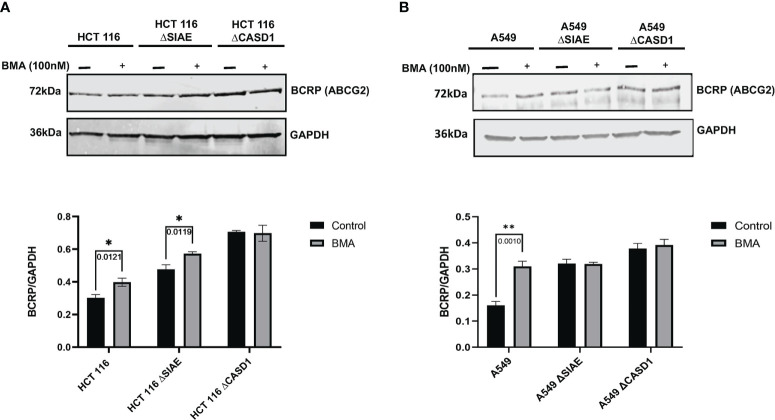
BCRP (ABCG2) expression in Wild type, Knockout (CASD1 and SIAE) cell lines following Bafilomycin A1 treatment (grey bars). Cells were treated with 100nM BMA, lysed and Immunoblot analysis was performed on whole cell lysate using BCRP-specific antibodies. GAPDH was used as loading control **(A)** Immunoblot analysis of whole cell lysates prepared from HCT 116 cell lines. Below is normalized Protein band density. **(B)** Immunoblot analysis of whole cell lysates prepared from A549 cell lines. Below is normalized Protein band density. Two-way ANOVA with Tukey’s posttest for multiple comparison, *P < 0.05 and **P < 0.01.

Protein degradation occurs in two major sites, namely lysosomes and proteasomes. To elucidate the role of the lysosomal pathway in the observed differences in BCRP protein level across the various cell lines (i.e., Wildtype and knockouts), cells were cultured in the presence or absence of 100 nM of potent lysosomal enzyme inhibitor, Bafilomycin A1 (BMA), and levels of BCRP in cell lysate determined *via* western blot. Whereas BCRP levels in CASD1 knockout cells remained significantly unchanged following BMA treatment in both cell lines, our results show significant increases in BCRP level for wild type and SIAE knockout cells of HCT 116 cells (p-values= 0.0121 and 0.0119 respectively) (**Figure 10A**). A significant increase in BCRP levels was also recorded for wild type of A549 cells (p-value= 0.0010), however no significant increase was seen in the SIAE knockout cells (p-value= 0.9879) (**Figure 10B**).

### CASD1 expression favors survival in lung adenocarcinoma

**Figure 11:**
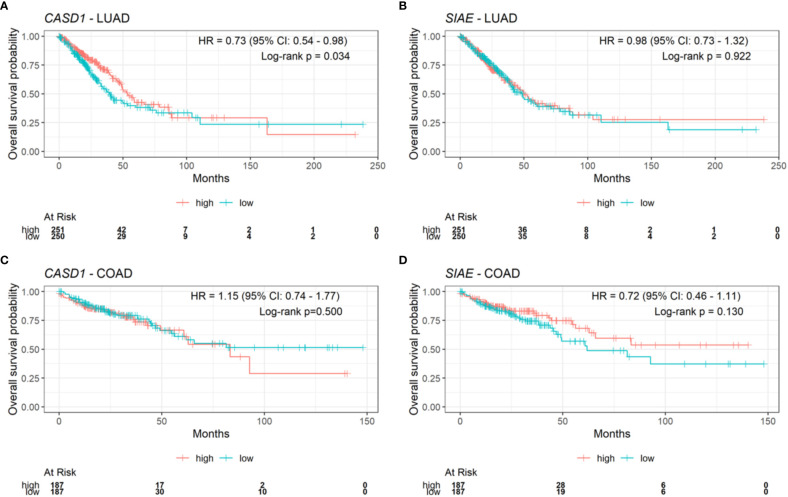
Kaplan-Meier plots for CASD1 and SIAE mRNA expression and overall survival in clinical lung adenocarcinoma (LUAD) and colon adenocarcinoma (COAD) samples. **(A)** CASD1 mRNA expression in LUAD samples. **(B)** CASD1 mRNA expression in COAD samples. **(C)** SIAE mRNA expression in LUAD samples. **(D)** SIAE mRNA expression in COAD samples. Cox proportional hazard regression hazard ratio, HR, (with 95% confidence interval, CI), and log-rank p value are annotated on each plot. Risk table is shown beneath each Kaplan-Meier plot. Samples were split into high (red) and low (blue) expression groups per gene using the median (high: >=median, low: <median).

After observing that CASD1 knockout promotes cell survival and proliferation in cancer cell lines, we investigated the clinical relevance of *CASD1* and *SIAE* expression in clinical samples by examining the relationship between overall patient survival and *CASD1* and *SIAE* mRNA expression in lung adenocarcinoma (LUAD) and colon adenocarcinoma (COAD) clinical samples from The Cancer Genome Atlas (TCGA). LUAD samples with high expression levels of *CASD1* (red line) were significantly associated with high patient survival rates (HR = 0.73, 95% CI: 0.54 – 0.98, log-rank p value = 0.034) compared to those with low expression (blue line) (**Figure 11A**). Median survival in the high and low *CASD1* expression groups are 53 months and 40 months respectively. This finding suggests that higher *CASD1* expression is a favorable marker of survival in LUAD. No significant association was observed in survival rates for (COAD) samples with high expression levels of *CASD1* compared to those with low expression (HR = 1.15, 95% CI: 0.74 – 1.77, log-rank p value = 0.500) (**Figure 11C**). Similarly, no significant associations were observed in *SIAE* expression and survival rates for patients with LUAD and COAD (log-rank p-value = 0.922 and 0.130 respectively) (**Figures 11B, D**).

## Discussion

The contribution of Sias in the process of multidrug resistance has remained unexplored despite the various roles Sias play in cancer progression and as viable targets for glycan related therapeutics. Sias have been implicated in the pathogenesis of a myriad of diseases including autoimmune disorders, inflammation, coronary artery disease, influenza infections, SARS-COV2 infections, cancer among others (40, 50–52). In cancers, Sia has been shown to mediate communication and interaction with receptors on immune cells *via* the sialic acid-Siglec pathway (35, 53).

Physiologically, more than 50 chemically distinct functional group modifications of Sia have been identified, among which the acetyl modification/variant is the most clinically studied (34). Cancer cells may differentially exploit these O-acetyl modifications in perpetuation of unique survival hallmarks such as immune cell evasion and aggressive phenotype development (39). For instance, Grabenstein et al. (35) in an extensive study, reported that Sia acetylation reduces engagement of cancer associated Siglecs and increases NK mediated cytotoxicity in colon and lung cancer cells. Comparative studies on colon-derived mucins have revealed over 50% acetylation of Sia in healthy tissues and predominantly high deacetylated Sia in cancerous tissues. Research has also shown that hypoacetylation of Sia on the tumor associated antigen, Sialyl Lewis X motifs, is the key alteration associated with metastatic colorectal cancers (54). Furthermore, high levels of 9-O acetyl sialylation of sialoglycoproteins have been shown to be distinctly expressed in human acute lymphoblastic leukemia (ALL) and used as diagnostic biomarker for the detection of pediatric ALL (55). It has also been touted to contribute to the survival and drug resistance characteristics observed in mouse and *in vitro* models of pre-B ALL (56). One of the ways in which cancer cells develop resistance toward chemotherapeutics is overexpression of sialyltransferases. ST6Gal-I sialyltransferase, an enzyme upregulated in numerous cancers has been reported to promote survival and resistance in ovarian, pancreatic, and colorectal cancers *via* hypersialylation of tumor necrosis factor receptor 1 (TNFR1) and Fas Receptor (FasR) death receptor (36, 37). Hypersialylation blocks receptor internalization and the formation of the death-inducing signaling complex (DISC), thereby disabling apoptotic signaling thus leading to cell survival (36, 37). Also, Ma et al. (38) reported overexpression of ST3GAL5 and ST8SIA4 sialyltransferases in drug resistant human acute myeloid leukemia (AML) cells relative to parental cancer cells in both *in vitro* and *in vivo* experimental models. Further studies revealed that altered levels of ST3GAL5 and ST8SIA4 correlated with high expression levels of P-gp and MRP1, suggesting a strong association between glycan sialylation and MDR.

In the current study, we explored the role of O-acetyl Sia modification in BCRP-mediated MDR. We employed lung cancer (A549) and colon cancer (HCT 116) cells whose sialate O-acetyl transferase (*CASD1*) gene and sialate O-acetyl esterase (*SIAE*) gene have been removed *via* CRISPR Cas 9 gene editing. The *CASD1* and *SIAE* genes encode key enzymes that respectively catalyze the addition and removal of acetyl groups to C-9 and/or C-7 positions of Sia (57, 58) (**Figure 1B**). The cells had been genotypically and phenotypically characterized in earlier studies (35, 40). For the purposes of this study, we further developed and characterized BCRP overexpressing HEK 293 cell to serve as positive control.

We first examined how these gene knockouts affected BCRP protein expression. Our results revealed high BCRP expression levels in the knockouts compared to the wild types of both cancer cells (**Figures 1C, D**). The CASD1 knockout cell lines (i.e., cells that lack O-acetyl Sia) in particular, demonstrated significantly higher levels of BCRP expression (**Figures 1C**). With respect to Sia modifications, prior studies have reported a positive correlation between Sialyl transferases, ST3Gal5 and ST8Sia4 expression and efflux proteins, PgP and MRP1 expression in human acute myeloid leukemia (38). Our data suggests that there is a strong correlation between dysregulation of O-acetylation in cells, particularly loss of CASD1, and BCRP expression. Colocalization experiments with membrane protein-specific probes are however needed to effectively establish whether altered Sialic acid acetylation disrupts BCRP trafficking (addressing) to the plasma membrane. Although all cells were imaged under same magnification (scale bar: 100um), a key observation made during microscopic analysis worth mentioning, is the cytological transformation (i.e., increase in size) of CASD1 knockout cells relative to the other cell variants. This may be attributed to its comparatively high expression of the BCRP efflux pump. Studies have shown that overexpression of membrane proteins reshapes membrane domain and induces elongation of the membrane, ultimately increasing cell size (59) Overexpression of BCRP confers resistance to several novel targeted molecules such as tyrosine kinase inhibitors as well as wide range of chemotherapeutic drugs including mitoxantrone, methotrexate and flavopiridol (60). In addition to its role of conferring resistance towards chemotherapeutic agents, BCRP also actively transports structurally diverse fluorescent compounds such as Hoechst 33342, BODIPY-prazosin, and pheophorbide A (9). Having identified the modulatory effects of O-acetyl Sia on BCRP expression, we next sought to probe how O-acetyl Sia modification affected efflux function of BCRP. Specifically, we elucidated Hoechst 33342 accumulation and cell sensitivity to Mitoxantrone in the wild type and knockout cells. Hoechst 33342(2’-[4-ethoxyphenyl]-5-[4-methyl-1-piperazinyl]-2,5’-bi-1H-benzimidazole trihydrochloride trihydrate) is a cell permeable dye that emits blue fluorescence when bound to double stranded DNA (61). Mitoxantrone on the other hand, is a chemotherapeutic agent that is clinically used to treat solid tumors, leukemias, and as an immune system modulator in multiple sclerosis. It functions as an anticancer agent by inhibiting topoisomerase II, an enzyme involved in DNA replication, chromosome condensation and segregation (62). This leads to increase in the incidence of double strand breaks and ultimately resulting in cell death. Our results showed significantly high levels of Hoechst 33342 accumulated in the nuclei of wildtype cell lines as compared to the knockout cell lines. The CASD1 knockout cell lines accumulated the least amount in their nuclei, hence recording the lowest fluorescent intensity. This may be attributed to the earlier reported variation in BCRP expression levels across the individual cell types. Generally, there is a positive correlation between efflux activity and levels of efflux proteins (i.e., BCRP) present in the cell. The wildtype cells extrude small amounts of Hoechst 33342 due to its low BCRP expression levels hence more of this fluorescent substrate accumulates in its nuclei. The knockout cells on the other hand, express high levels of BCRP hence they extrude large amounts of Hoechst 33342, ultimately resulting in the observed low intracellular (nuclei) levels, especially in CASD1 knockout cells. These variations in BCRP efflux activity also accounts for the observed responses of the various cell types towards the chemotherapeutic drug, mitoxantrone. Wildtype of both experimented cancer cells were observed to be more sensitive to mitoxantrone. This may be due to their inability to extrude high amounts of mitoxantrone because of their inherently low BCRP levels. Thus, high amount of this cytotoxic agent is retained intracellularly, leading to a reduction in cell viability. Conversely, the CASD1 knockout cells were observed to be more refractory (2- and 3-fold resistance for colon and lung cancer cells respectively) to mitoxantrone. The high efflux activity culminating from high BCRP levels in CASD1 knockout cells may account for this observation. These cells minimize intracellular levels of mitoxantrone and its associated cytotoxic effect by actively pumping out relatively high amounts of mitoxantrone out of the cells. Our data therefore suggests that the absence of O-acetyl Sia (Deacetylated Sia) confers drug resistance characteristics in lung and colon cancer cells. This observation is in contrast with earlier studies conducted by Parameswaran et al. (56) who reported a strong correlation between the presence of 9-O acetyl Sia and vincristine or nilotinib drug-resistant ALL cells. These contradictory observations suggest that the relationship between O-acetyl Sia modification and MDR may be cell or cancer specific. Also, since vincristine is not a substrate of BCRP, it suggests BCRP may not be involved in the earlier studies involving ALL cells but rather P-glycoprotein (PgP) efflux pump. Both nilotinib and vincristine are substrates of PgP (63) thus, we speculate that the role of O-acetyl Sia modification in MDR may also be efflux protein specific.

We further investigated whether O-acetyl Sia mediated-BCRP modulation, promotes phenotypic cell survival characteristics such as enhanced cell proliferation. Our results showed significantly high growth rate in CASD1 knockout lung cancer cells compared to wildtype and SIAE knockout cell lines. A similar pattern was recorded for the colon cancer cells albeit differences in growth rate were not significant. This observation is consistent with earlier reports that implicate Sia hypo/deacetylation as key alteration associated with aggressive and metastatic colorectal cancers (54). BCRP is also known to be a stem cell marker, whose expression in cancer cells is driven by metabolic and signaling pathways that confer multiple mechanisms of drug resistance, invasiveness (aggressiveness), and self-renewal (64) hence confirming the observed high proliferative ability of the BCRP-overexpressing CASD1 knockout cells.

We further elucidated the expression levels of BcL-2 and Poly-ADP Ribose Polymerase (PARP), two proteins that have been shown to be key drivers of cell proliferation and cell survival. BcL-2 family of proteins are key regulator of apoptosis that primarily function by either inhibiting or promoting cell death. The fate of a cell is dependent on the balance between pro-apoptotic and pro-survival members of the BcL-2 family. Pro-apoptotic members of this family such as Bax and Bak promote cell death by direct binding interaction that disrupts mitochondrial outer membrane potential leading to irreversible release of intermembrane space-bound protein cytochrome C, subsequent caspase activation and apoptosis (65). Pro-survival BcL-2 family members such as BcL-2 and Bcl-xL on the other hand, prevent apoptosis by inhibiting mitochondrial outer membrane depolarization and promoting cell survival and proliferation (66). Our results revealed consistently significant high levels of the pro-survival protein BcL-2 in CASD1 knockout cells compared to the wild type and SIAE knockout cell lines for the lung cancers and wild type for the colon cancers. This once again corroborates the significantly high proliferation observed earlier in the CASD1 knockout cells. PARP 1, a 116kDa protein, belonging to the PARP superfamily, is a crucial protein involved in ensuring cell survival. Physiologically, full or active PARP 1 is known for its involvement in DNA repair processes (67). Enzymatic cleavage of PARP 1 by active Caspase 3 results in 2 inactive fragments that are unable to facilitate DNA repair leading to genomic instability and ultimately cell death. Evidence from several studies have reported upregulation of PARP activity in some cancer types. For instance, a study conducted on hepatocellular carcinoma patients showed significantly increased levels of PARP in tumor tissues than adjacent non-tumorous tissues (68). Also, strikingly high PARP 1 mRNA levels have been reported in triple negative breast cancer tumors and tumors of the endometrium, lung, ovary, and skin (69). In comparison to the wild type and SIAE knockout cells, our results showed significantly low levels of 25kDa cleaved/inactive PARP1 fragments in the CASD1 knockout lung and colon cancer cells when challenged with mitoxantrone. This observation may be attributed to the rapid efflux of mitoxantrone by CASD1 cells resulting in significant reduction of the cytotoxic concentration required to stimulate the protein mediators of the apoptosis cascade which includes Cleaved PARP1 (44). This data also confirms and corroborates our earlier findings that sialic acid deacetylation promotes cell survival in lung and colon cancer cells.

Gene expression modulatory factors and post-translational modification check points at the endoplasmic reticulum (ER) and endosome-lysosome govern the relative expression levels and stability of most proteins (70). Thus, to better understand the factor responsible for the varied protein expression levels and function of BCRP, we determined the levels of BCRP mRNA in each of the cell variants. CASD1 knockout cells significantly expressed high levels of BCRP mRNA relative to wild type and SIAE knockouts of all cell types. This finding support/confirm the BCRP protein expression data, suggesting that deacetylation of sialic acid acids alters (increases) BCRP mRNA transcript levels and consequently upregulates level of expressed proteins. We also probed for the involvement of the Lysosomal Protein degradation pathway in this phenomenon. CASD1 is localized at the trans Golgi membrane where it modifies terminal sialic acids on glycoproteins before they are trafficked to the plasma membrane. An earlier study reported that majority of plasma-membrane bound glycoproteins with O-acetyl functional group modifications are retained in the Golgi compartment (i.e., 7,9-O- and 9-O-Ac) whereas only some (9-O-Ac) are transported to the cell surface (40, 71). After remaining in the plasma membrane domain for a short period, these unstable modified glycoproteins are recycled and degraded *via* the endosome-lysosome pathway. We therefore blocked protein degradation *via* the lysosome by treating all cell variants with Bafilomycin A1 (BMA). BMA is a macrolide antibiotic that prevents maturation of autophagic vacuoles by inhibiting fusion between autophagosomes and lysosomes (72). It also serves as a specific inhibitor of vacuolar H ^+^ ATPase (V-ATPase) causing alkalinization of the lysosome lumen, impairing lysosomal enzyme function and subsequently leading to cellular accumulation of lysosome-bound proteins (73). We observed significantly increased levels of BCRP in BMA-treated cells in the wildtype and SIAE knockout cells in both lung and colon cancer cell lines. However, no significant increase was recorded in BCRP level for BMA-treated CASD1 knockout cancer cells suggesting that lysosomal degradation pathway may also be responsible for modulating levels of acetyl modified glycoproteins including BCRP. Consistent with our experimental findings that CASD1 knockout confers resistance to mitoxantrone and promotes cell survival, we also found that CASD1 expression favors survival in clinical lung Adenocarcinoma (LUAD) samples. This implies that CASD1-mediated sialic acid acetylation may represent a potential therapeutic target in LUAD requiring further studies to understand how the mechanism and modifiers of this process counteracts BCRP-mediated drug resistance.

Taken together, our results highlight the crucial role O-acetyl Sia modification play in cancer related MDR. Specifically, this study provides empirical evidence that modulation of acetyl Sia (specifically deacetylated Sia) upregulates BCRP expression and promote survival in lung and colon cancer cell lines. Clinical data also validated that in patients with LUAD, lower levels of CASD1 and thus less O-acetyl Sia expression had lower survival rates than those patients with high CASD1 expression. Findings from this study may have relevance to a broader spectrum of malignancies and provide a promising avenue for future MDR-circumventing therapeutics. Further studies are however warranted to explore the effect of acetyl Sia modulation on other cancers as well as other relevant MDR efflux proteins such as Pgp and MRP1.

## Data availability statement

The datasets presented in this study can be found in online repositories. The names of the repository/repositories and accession number(s) can be found in the **Supplementary Material**.

## Ethics statement

This study used *CASD1* and *SIAE* mRNA expression and overall survival data from de-identified clinical lung adenocarcinoma and clinical colon adenocarcinoma samples publicly available online (https://www.cbioportal.org/). These data were derived from previously approved human cancer genomics study The Cancer Genome Atlas (TCGA, accession numbers: PRJNA74949, PRJNA41443).

## Author contributions

RW-C ad IS contributed to conception and design of the study. IT performed experiments, data analysis and wrote the first draft of the manuscript. SA performed clinical data analysis and wrote sections of the manuscript. HB performed experiment and data analysis. CP generated A549 Lung cancer knockout cell lines. All authors contributed to manuscript revision, read, and approved the submitted version. All authors contributed to the article and approved the submitted version.
