# Supplementary figures and images for: Early in vitro evidence indicates that deacetylated sialic acids modulate multi-drug resistance in colon and lung cancers via breast cancer resistance protein
Source: Front Oncol. 2023 Jun 12;13:1145333. doi: 10.3389/fonc.2023.1145333 (PMC10291187; doi:10.3389/fonc.2023.1145333)

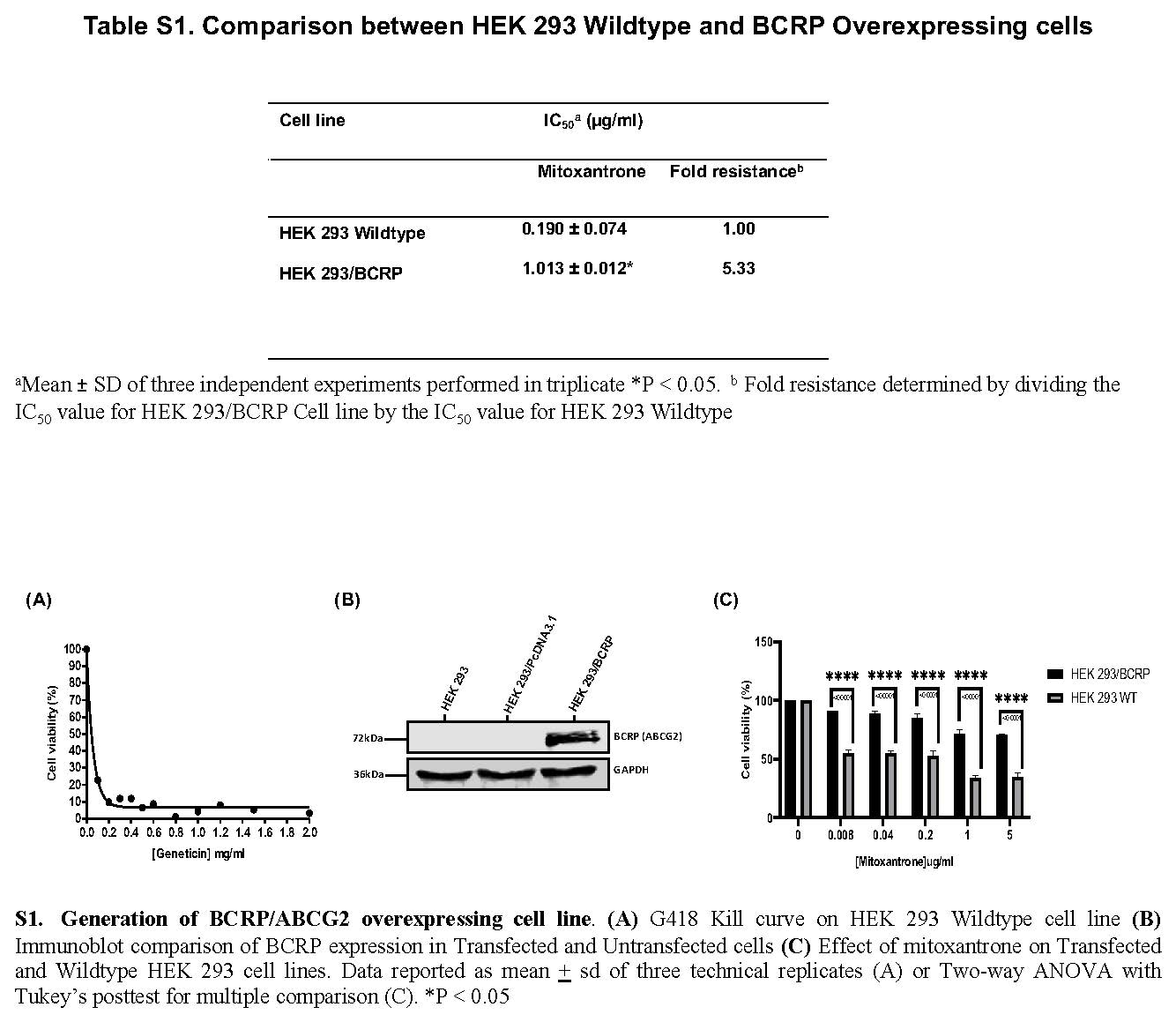

Supplement: Supplementary file 1 [file Image_1.jpg]
